# Expression levels of the tetratricopeptide repeat protein gene *ttc39b* covary with carotenoid-based skin colour in cichlid fish

**DOI:** 10.1098/rsbl.2020.0629

**Published:** 2020-11-25

**Authors:** Ehsan Pashay Ahi, Laurène A. Lecaudey, Angelika Ziegelbecker, Oliver Steiner, Walter Goessler, Kristina M. Sefc

**Affiliations:** 1 Institute of Biology, University of Graz, Universitätsplatz 2, A-8010 Graz, Austria; 2 Department of Comparative Physiology, Uppsala University, Norbyvägen 18A, SE-75 236 Uppsala, Sweden; 3 Department of Natural History, NTNU University Museum, Norwegian University of Science and Technology, NO-7491 Trondheim, Norway; 4 Institute of Chemistry, University of Graz, Universitätsplatz 1, A-8010 Graz, Austria

**Keywords:** carotenoids, colour genes, cichlidae, animal body colouration, colour polymorphism, comparative transcriptomics

## Abstract

Carotenoid pigments play a major role in animal body colouration, generating strong interest in the genes involved in the metabolic processes that lead from their dietary uptake to their storage in the integument. Here, we used RNA sequencing (RNA-Seq) to test for differentially expressed genes in a taxonomically replicated design using three pairs of related cichlid fish taxa from the genera *Tropheus* and *Aulonocara*. Within each pair, taxa differed in terms of red and yellow body colouration, and high‐performance liquid chromatography (HPLC) analyses of skin extracts revealed different carotenoid profiles and concentrations across the studied taxa. Five genes were differentially expressed in all three yellow–red skin contrasts (*dhrsx*, *nlrc3*, *tcaf2*, *urah* and *ttc39b*), but only the tetratricopeptide repeat protein-coding gene *ttc39b*, whose gene product is linked to mammalian lipid metabolism, was consistently expressed more highly in the red skin samples. The RNA-Seq results were confirmed by quantitative PCR. We propose *ttc39b* as a compelling candidate gene for variation in animal carotenoid colouration. Since differential expression of *ttc39b* was correlated with the presence/absence of yellow carotenoids in a previous study, we suggest that *ttc39b* is more likely associated with the concentration of total carotenoids than with the metabolic formation of red carotenoids.

## 1. Introduction

Much of the colour diversity in the animal kingdom is produced by carotenoid pigments. Animals acquire carotenoids from their diet, modify them enzymatically and deposit taxon- and tissue-specific mixtures of carotenoids in integumentary tissues such as skin and feathers [1]. The resulting carotenoid-based body colour varies with both the concentration and types of the integumentary carotenoids. Carotenoid body colouration can therefore be influenced by diet as well as genetic factors that control the uptake, storage and biochemical transformations of the pigments [1]. To date, relatively few genes that affect carotenoid colouration have been identified in vertebrates [1], including, among others, the ketolase CYP2J19 [2], the carotenoid cleavage enzyme BCO2 [3] and the lipoprotein receptor SCARB1 [4]. While some carotenoid colour genes are restricted to particular vertebrate lineages (e.g. [5]), others are more widely conserved across taxonomic groups (e.g. [3,4,6–8]).

Carotenoid pigments are also involved in the outstanding body colour diversity among cichlid fish in African and South American waters [9,10]. In fish skin, carotenoids are stored in carotenoid droplets that are located within erythrophores (red pigment cells) and xanthophores (yellow pigment cells). Carotenoid droplets are structurally homologous to lipid droplets [11], and hydroxylated carotenoids are typically esterified with fatty acids to increase their liposolubility and facilitate carotenoid droplet formation [12]. Recent studies hint at similarities in genetic mechanisms controlling the metabolism and intracellular storage of carotenoids and neutral lipids [11,13].

**Figure 1.**
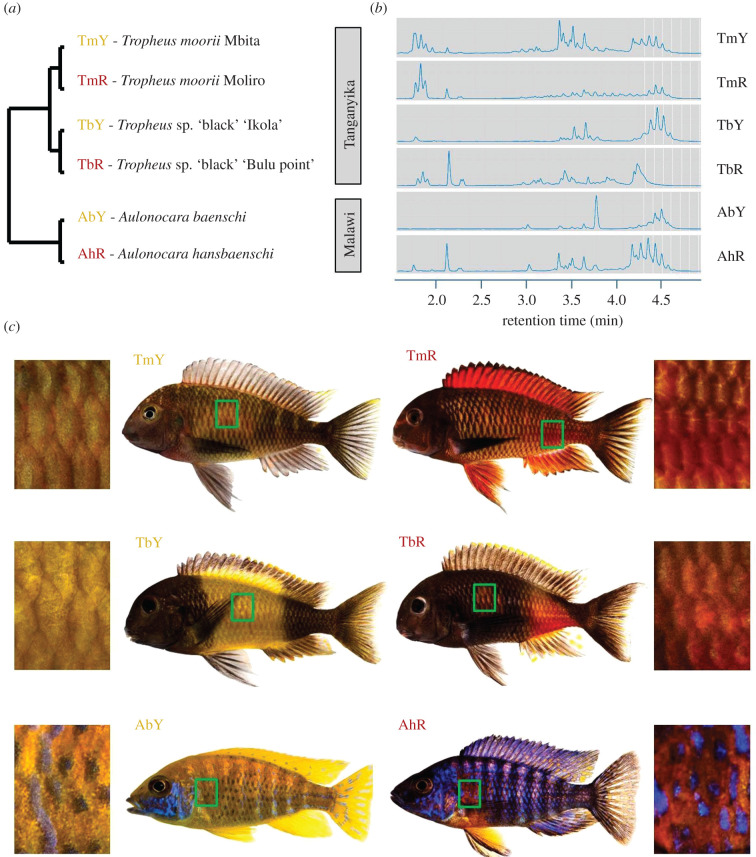
Cichlid taxa and skin regions analysed in the present study. (*a*) Schematic illustration of the phylogenetic relationships between the investigated cichlid taxa. (*b*) Ultra‐high‐performance liquid chromatography (UHPLC) chromatograms of carotenoid extracts of one typical skin sample per colour variant. Peaks after 2.5 min correspond to esterified carotenoids (except for free beta carotene at 3.8 min in *A. baenschi*). For a larger view, see electronic supplementary material, figure S1. (*c*) The skin regions sampled for paired comparisons between cichlid colour variants are marked by green boxes on the fish bodies and depicted in macroscopic photographs. Taxon codes as shown in (*a*).

In the present study, we used RNA sequencing (RNA-Seq) to screen for genes associated with carotenoid colour differentiation among three pairs of related cichlid taxa, which differ in terms of red and yellow carotenoid-based body colouration (figure 1). This design allowed us to test for consistent differential gene expression across taxonomically replicated skin colour contrasts. We detected the elevated expression of a tetratricopeptide repeat (TPR) protein, *ttc39b*, in the red skin samples in each contrast. A previous study associated *ttc39b* with avian bill colour polymorphism [14], and our findings contribute to emerging evidence for a function of *ttc39b* in carotenoid colouration across vertebrate classes.

## 2. Material and methods

We sampled yellow and red skin patches from three pairs of related cichlid taxa (*n* = 6 males per colour variant) as indicated in figure 1. The taxon pairs (yellow versus red) are (i) *Tropheus moorii* ‘Mbita' versus *T. moorii* ‘Moliro', (ii) *T.* sp. ‘black' ‘Ikola' versus *T.* sp. ‘black' ‘Bulu Point', all four from Lake Tanganyika and (iii) *Aulonocara baenschi* versus *A. hansbaenschi* ‘Red Flush’ from Lake Malawi (figure 1). The taxa are abbreviated as TmY, TmR, TbY, TbR, AbY and AhR (figure 1) in the text and figures. Divergence times between the taxa within each pair are on the scale of 100 000 to 1 million years, whereas the two *Tropheus* population pairs (*T. moorii* and *T.* sp. ‘black') diverged 1–2 Mya, and divergence between *Tropheus* and *Aulonocara* is 5 Mya [15] (figure 1). The adult, captive-bred fish were obtained from the aquarium trade. For a minimum of four weeks prior to the experiment, the fish were kept in our aquarium facility and fed identical flake food diets providing a mixture of algal, animal and plant carotenoids (Spirulina Super Forte 36, Tropical). Before dissection, fish were sacrificed in a solution of 1 g MS-222 per 1 L water. Scales were removed and discarded, and the skin tissue samples were immediately transferred into RNAlater (Qiagen) and stored at −20°C.

Details on laboratory and analysis protocols are given in the electronic supplementary material. Briefly, the extraction of total RNA and RNA-Seq library preparation were performed as described [13]. Following RNA extraction with the ReliaPrep™ RNA Tissue Miniprep System Kit (Promega), the RNA integrity number (RIN) of extracts was checked in a R6 K ScreenTape System on an Agilent 2200 TapeStation (Agilent Technologies, Waldbronn, Germany) and exceeded 7 in all samples. Libraries were prepared with the Standard TruSeq Stranded mRNA Sample Prep Kit (Illumina) using 1500 ng RNA, checked for quality on a D1000 ScreenTape on an Agilent 2200 TapeStation and sequenced by the NGS Facility at Vienna Biocenter Core Facilities (VBCF, Austria) in order to generate 125 bp paired-end reads (7.6–17.0 million raw reads per sample). Raw reads were demultiplexed by the sequencing facility and checked for quality using the FASTQC tool [16]. Trimmomatic software [17] was used to trim the dataset to reads with a phred +33 quality score of at least 34 for all bases and a minimum length of 50 bp (7.6–16.8 million trimmed reads per sample; electronic supplementary material, table S1). Sequence reads are available from the NCBI sequence read archive (SRA) under the accession number PRJNA658843. Gene expression analysis and gene annotation were carried out as described [13].

First-strand cDNA for quantitative PCR (qPCR) was synthesized from 500 ng RNA of each skin sample extract using the high capacity cDNA reverse transcription kit (Applied Biosystems) and diluted 1 + 3 for the subsequent qPCR reactions. Primer design and the qPCR protocol are described in the electronic supplementary material. Relative expression levels (RQ) were determined by the 2−ΔΔCq method [18] using geometric means of the Cq values of two reference genes, *clf2* and *cct3*, to normalize Cq values of the target genes. Log-transformed RQ values were compared between red and yellow skin samples using *t*-tests (electronic supplementary material, tables S2–S4).

Associations between gene expression levels and skin colour (yellow, red) were tested by phylogenetically controlled ANOVAs using the R-package *geiger* [19]. The phylogeny reflected the above divergence time estimates, with minimal divergence within taxa.

Integumentary carotenoids were extracted from some of the same fish as used for RNA-Seq (*n* = 3 males per colour variant), with skin samples taken from the same body region on the other side of the fish. Samples were extracted twice in a solution of acetone with butylated hydroxytoluene (BHT, 1 g l^−1^) and analysed on an Agilent 1290 UHPLC System with an Agilent Zorbax Eclipse Plus C18 (2.1 × 50 mm, 1.8 µm Rapid resolution HD). Signals were recorded at 480 nm. Integrated peak areas from the two consecutive extractions were summed for the assessment of signal strength, relative to skin sample fresh weight, as a proxy of the relative carotenoid content in the skin sample.

## 3. Results

**Figure 2.**
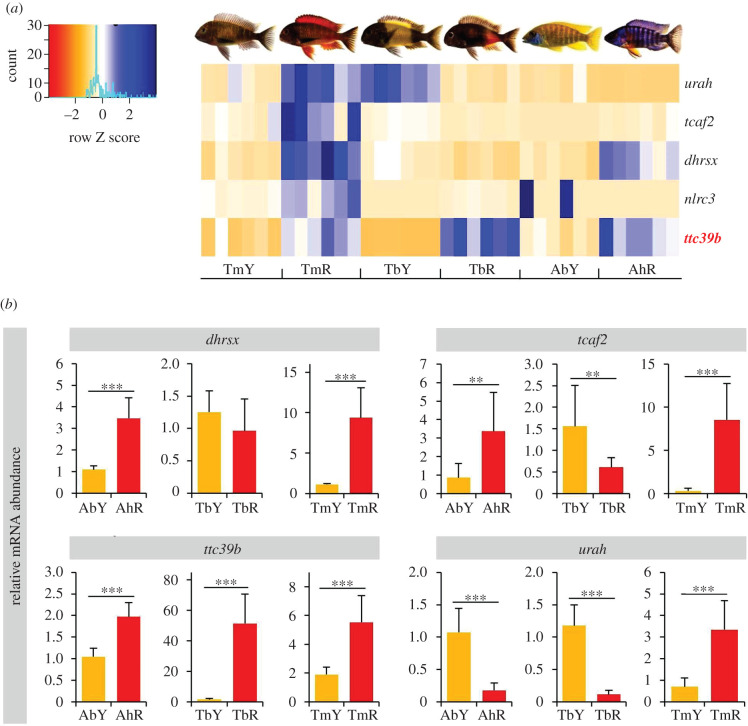
Differential gene expression. (*a*) Heatmap showing differential gene expression between yellow and red skin samples. Blue and orange shadings represent higher and lower relative expression levels, respectively. (*b*) qPCR validation of RNA-Seq expression patterns. Bars represent means and standard deviations of RQ in six biological replicates. Asterisks indicate significant differences in expression levels between the dorsal and ventral samples in within-population comparisons (*t*-tests; ***, *p* < 0.001; **, *p* < 0.01). Taxon codes as in figure 1*a*.

High–performance liquid chromatography (HPLC) analysis of skin extracts confirmed the presence of free and esterified carotenoids in both yellow and red coloured cichlid variants. Carotenoid profiles varied between colour variants, and integrated peak areas were larger in the red skin samples compared to the contrasted yellow variants (figure 1*b*; electronic supplementary material, figure S1 and S2 and table S5). Among the differentially expressed genes in each taxon pair, only five genes showed significant expression differences between yellow and red skin samples in all three colour contrasts: *dhrsx*, *nlrc3*, *tcaf2*, *urah* and *ttc39b* (electronic supplementary material, figure S3 and table S6). Of these, only *ttc39b* expression was consistently regulated in each taxon pair, showing significantly higher expression levels in the red skin tissues relative to the yellow tissues (figure 2*a*). Two genes, *dhrsx* and *tcaf2*, showed congruent expression patterns with TmY < TmR, TbY > TbR and AbY < AhR. Differential expression of *urah* followed the pattern TmY < TmR, TbY > TbR and AbY > AhR, and that of *nlrc3* was TmY < TmR, TbY > TbR and AbY > AhR (figure 2*a*).

The RNA-Seq results were validated by qPCR analysis of *ttc39b*, *dhrsx*, *tcaf2* and *urah* expression levels (*nlrc1* was not analysed by qPCR). In each cichlid pair, expression levels of *ttc39b* were significantly higher in the red compared to the yellow skin (figure 2*b*). Similarly, qPCR confirmed the RNA-Seq results for the other three genes, except for a non-significant result for *dhrsx* in the comparison between TbY and TbR (figure 2*b*; electronic supplementary material, table S4).

In accordance with the prevalence of taxon pair-specific expression patterns, phylogenetically controlled ANOVAs across all taxa revealed no significant associations between skin sample colour and expression levels of *nlrc3*, *tcaf2* and *urah.* By contrast, expression levels of *dhrsx* (*F*_1,34_ = 20.02, *p* = 0.030 for qPCR data) and *ttc39b* (*F*_1,34_ = 65.02, *p* = 0.005 for RNA-Seq data; *F*_1,34_ = 22.96, *p* = 0.026 for qPCR data) showed significant covariation with colour across taxa (all results shown in electronic supplementary material, table S7).

## 4. Discussion

We report consistent differential expression of *ttc39b* in the skin of three pairs of closely related cichlid fish taxa, which differed in red versus yellow body colouration and skin carotenoid content. Since our experimental fish had been fed identical diets, differences in integumentary carotenoid content must have been produced by metabolic processes. Currently, there is no functional evidence for a connection between *ttc39b* expression and carotenoid colouration, but the role of its gene product in cholesterol and lipid metabolism [20] suggests possible links via the parallels between triglyceride and carotenoid metabolism [11,13]. The mammalian TTC39B protein contains three consecutive TPR motifs, suggesting its function is as a scaffold protein to mediate protein–protein interactions and the assembly of multiprotein complexes of HDL-regulating proteins [21,22]. The expression of *ttc39b* interferes with Liver X Receptor (LXR) signalling [22], which regulates cholesterol homeostasis [23] and may be associated with carotenoid uptake via regulation of *npc1l1* expression [23,24]. The association of *ttc39b* with changes in human blood lipoprotein levels [21] and with LXR signalling [22] might point towards a possible effect of *ttc39b* on carotenoid uptake and storage.

Differential expression of *ttc39b* has been detected in previous RNA-Seq studies concerned with body colour variation. These studies reported the elevated expression of *ttc39b* in the orange compared to the white skin regions of one of two examined clownfish species [25], in the skin of yellow compared to blue coloured morphs of a cichlid fish [26] and in yellow compared to white skin regions on the body of another cichlid fish [13]. Furthermore, avian TTC39B was found to be located on a Z chromosomal region that co-segregates with yellow–red bill colour polymorphism in an estrildid finch [14]. Collated across the repeated, but rather unheeded signals of *ttc39b* from previous studies, and combining it with the strong signal of *ttc39b* in our dataset, we propose *ttc39b* as a compelling, novel candidate gene for carotenoid colouration.

The association of *ttc39b* with yellow–red colour variation that we observed here and that has previously been described in the estrildid finch [14] hints toward a role of *ttc39b* in the metabolic formation of red carotenoids. This interpretation is also compatible with increased expression of *ttc39b* in the reddish-orange clownfish skin [25]. It is, however, contradicted by the elevated expression of *ttc39b* in the yellow compared to non-carotenoid coloured skin detected in other fish studies [13,26], in particular since the coloured skin samples in [13] were dominated by yellow carotenoids. That study also verified that the differential expression of *ttc39b* was not related to variation in integumentary triglyceride content. We therefore consider it possible that *ttc39b* expression covaries with integumentary carotenoid concentration rather than composition in this and the previous studies. Quantitative comparisons of total carotenoid concentrations are confounded when samples contain different types of carotenoids, as is the case in our study (electronic supplementary material, figure S1). Furthermore, although all skin samples were treated equally, extraction efficiencies may nevertheless have varied among samples. Nonetheless, a crude approximation by HPLC peak area integration indeed points towards higher carotenoid concentrations in the red than in the yellow skin samples across the investigated cichlid variants, congruent with the variation in *ttc39b* expression levels (electronic supplementary material, figure S2).

The differences among the HPLC chromatograms of the skin carotenoid extracts imply different biochemical backgrounds of the yellow–red colour contrasts represented by the three taxon pairs. This conforms with the gene expression data, which—except for *ttc39b*—also demonstrated taxon pair-specific patterns of differential gene expression. Together, these data hint at variation in the molecular mechanisms behind the expression of carotenoid colour diversity in cichlid fish.

Another tetratricopeptide repeat protein, RCP2, was recently found to regulate carotenoid accumulation in the floral tissues of monkeyflowers [27]. While TPR proteins participate in diverse eukaryotic cell processes through mediating versatile protein–protein interactions [28], the intriguing possibility of molecular parallels between plant and animal colouration calls for further study. Based on existing evidence, the implication of *ttc39b* in the carotenoid-based colouration of both birds and fishes suggests a conserved function of the gene in vertebrate colouration.

## Supplementary Material

Electronic supplementary material file 1

## Supplementary Material

Electronic supplementary material file 2

## Data accessibility

The datasets supporting the conclusions of this article are available as follows: sequence reads from the RNA-Seq experiment are available from the NCBI sequence read archive under the accession number PRJNA658843 (https://www.ncbi.nlm.nih.gov/bioproject/658843). Data from the RNA-Seq and qPCR experiments and the carotenoid HPLC analysis are provided in the electronic supplementary material.
