# Supplementary material for: Expression levels of the tetratricopeptide repeat protein gene ttc39b covary with carotenoid-based skin colour in cichlid fish
Source: Biol Lett. 2020 Nov 25;16(11):20200629. doi: 10.1098/rsbl.2020.0629 (PMC7728679; doi:10.1098/rsbl.2020.0629)
Supplement: Electronic supplementary material file 1 [file rsbl20200629supp1.docx]

**Electronic Supplementary Material for “Expression levels of the tetratricopeptide repeat protein gene *ttc39b* co-vary with carotenoid-based skin color in cichlid fish,”**

Ehsan Pashay Ahi^1,2^, Laurène A. Lecaudey^1,3^, Angelika Ziegelbecker^1^, Oliver Steiner^4^, Walter Goessler^4^, Kristina M. Sefc^1*^

1. Institute of Biology, University of Graz, Universitätsplatz 2, A-8010 Graz, Austria

2. Department of Comparative Physiology, Uppsala University, Norbyvägen 18A, SE-75 236 Uppsala, Sweden

3. Department of Natural History, NTNU University Museum, Norwegian University of Science and Technology, NO-7491 Trondheim, Norway

4. Institute of Chemistry, University of Graz, Universitätsplatz 1, A-8010 Graz, Austria

*Corresponding author: [kristina.sefc@uni-graz.at](mailto:kristina.sefc@uni-graz.at)

**Supplementary material and methods:**

**RNA extraction**

Skin samples were immersed into 250 µl of LBA buffer mixed with the recommended volume of 1-thioglycerol and 1.4 mm ceramic spheres and homogenized using a FastPrep-24 Instrument (MP Biomedicals, CA, USA). Total RNA was extracted using the ReliaPrep™ RNA Tissue Miniprep System Kit (Promega) according to the manufacturer's protocol for fibrous tissues. The protocol includes a column-based genomic DNA removal step together with several purification steps without the need for phenol-chloroform phase separation and ethanol precipitation steps. The extracted RNA was eluted in 30 µl nuclease-free water and quantified with a Nanophotometer (IMPLEN GmbH, Munich, Germany). The quality of all RNA extracts was checked in a R6K ScreenTape System on an Agilent 2200 TapeStation (Agilent Technologies) and they all had RNA integrity number (RIN) above 7.

**RNA-Seq library preparation and transcriptome comparisons**

For transcriptome sequencing, we performed library preparation according to the protocol of the Standard TruSeq Stranded mRNA Sample Prep Kit (Illumina) using 1500 ng RNA per sample. We checked the quality of the libraries on D1000 ScreenTape on an Agilent 2200 TapeStation (Agilent Technologies). The libraries were diluted to an optimal quantity required for sequencing and sequenced by the NGS Facility at Vienna Biocenter Core Facilities (VBCF, Austria) in order to generate 125bp paired-end reads. Each sample yielded between 7.6-17.0 million reads. After de-multiplexing the barcoded reads by the sequencing facility, a quality control step was conducted on the raw reads for each sample using the FASTQC tool (Andrews, 2012). The low quality reads for each sample were removed based on a recommended standard quality trimming step through the Trimmomatic software (Bolger, Lohse & Usadel, 2014). Only reads with a phred +33 quality score of at least 34 for all bases, and a minimum length of 50 bp were retained for downstream analyses (7.6-16.8 million trimmed reads per sample; Table S1). Sequence reads are available from the NCBI sequence read archive (SRA) under the accession number PRJNA658843.

Based on the transcriptome assembly, the transcript abundances were quantified for each sample using Kallisto, a tool integrated in the Trinity software package, in order to obtain sample-specific expression levels of each transcript at gene level [1]. Transcripts per million transcripts (TPM), generated by Kallisto, was used as gene expression unit for downstream analysis. To normalize the data across samples, the weighted trimmed mean of the log expression ratios (TMM) was used. Gene expression levels were then compared between the yellow and red skin samples within each of the three taxon pairs. We used Trinity to construct normalized expression matrices of all samples and the edgeR package, from the R Bioconductor software (R version 3.4.4, R Development Core Team 2018), to detect differentially expressed transcripts in each of the three investigated taxon pairs [2–5]. Significantly differentially expressed genes were extracted from the results, using a false-discovery rate (FDR) cutoff of 0.1 [6] and a minimum of 1 fold-change (two times relative expression difference).

To annotate the genes, we first used TransDecoder software (http://transdecoder.github.io) to identify ORFs with complete coding sequences. TransDecoder identifies candidate protein-coding regions based on nucleotide composition through detection of a minimum length ORF per gene, computation of a log-likelihood score for each ORF and reporting the longest ORFs per gene [7]. To maximize sensitivity for capturing ORFs with functional significance, we scanned all ORFs for homology through BLAST tool [8] against coding sequences (CDS) of Nile tilapia and two other distant teleost fish species *Danio rerio* and *Gasterosteus aculeatus* for further confirmation. We used the following NCBI CDS annotations: assembly ASM185804v2 (ID: GCF_001858045.1) for the Nile tilapia, assembly GRCz10 (ID: GCF_000002035.5) for *Danio rerio* and assembly CriGri_1.0 (ID: GCF_000223135.1) for *Gasterosteus aculeatus*.

**Real time quantitative PCR (qPCR)**

We selected nine candidate reference genes from the transcriptome data as described in [9,10]. In brief, for each transcriptome comparison, we identified the genes with no expression difference (FDR = 1) between the skin tissues and ranked them according to their expression level to obtain the top 100 genes with highest expression.  Then, we ranked these genes by their coefficient of variation (CV of expression levels) across biological replicates. The top nine genes shared between the three comparisons were selected as candidate reference genes. Based on the qPCR assays, the candidate reference genes were ranked according to expression stability by three different algorithms, BestKeeper [11], NormFinder [12] and geNorm [13]. Based on these results (Table S7), *clf2* and *cct3* were selected as reference genes to normalize expression levels of the target genes.

To design qPCR primers for the two reference genes and the five target genes, we constructed an alignment of the assembled sequence of each gene and its homologous sequences in other African cichlids including three species of the tribe Tilapiini (*O. aureus*, *O. mossambicus* and *O. niloticus*), one species of Lamprologini (*Neolamprologus brichardi*), one species of Ectodini (*Callochromis macrops*), and four species of Haplochromini (*Maylandia zebra*, *Pundamilia nyererei*, *Ctenochromis horeii* and *Astatotilapia burtoni*) [14–16]. This allowed us to find conserved sequence regions across the species and at the exon junctions (using CLC Genomic Workbench, CLC Bio, Denmark and the annotated genome of *Oreochromis_niloticus* in the Ensembl database, <http://www.ensembl.org>). All primers were designed to produce a short amplicons (<250 bp) with the help of Primer Express 3.0 (Applied Biosystems, CA, USA) and their structural configurations were evaluated using OligoAnalyzer 3.1 (Integrated DNA Technology) (Table S2).

500 ng RNA of each skin sample extract (see section *RNA extraction*) were used for first strand cDNA synthesis using the High Capacity cDNA Reverse Transcription kit (Applied Biosystems) according to the manufacturer's protocol. cDNA was then diluted 1+3 (v/v) for the subsequent qPCR reactions. qPCR reactions followed the protocol provided by Maxima SYBR Green/ROX qPCR Master Mix (2X) (Thermo Fisher Scientific, Germany) and the guidelines for optimal experimental set-up [17]. The qPCR program started with a 2 min hold at 50°C, a 10 min hold at 95°C, followed by 40 cycles of 15 sec at 95°C and 1 min at 60°C, and finally a dissociation step at 60°C – 95°C. The primer efficiencies (E values) were calculated by the LinRegPCR v11.0 programme [18] (Table S2).

The geometric means of the Cq values of the two reference genes were used to normalize Cq values of target genes in each sample (ΔCq _target_ = Cq _target_ – Cq _reference_). For each gene, the normalized expression in one arbitrary sample of the dorsal bar region of *T. duboisi* “Maswa” was set as calibrator for the calculation of ΔΔCq values (ΔCq target – ΔCq calibrator). Relative expression levels (RQ) were determined by the 2−ΔΔCq method [19]. The log-transformed RQ values were used for t-tests to infer statistically significant differences between differently colored skin samples.

Associations between gene expression levels (TMM normalized RNS-Seq read counts, Table S9, and log-transformed RQ values, table S3) and skin colour (as a binary variable with levels ‘yellow’ and ‘red’) across all samples were tested by phylogenetically controlled ANOVAs using the function *aov.phylo* in the R-package *geiger* (REF), with 10,000 simulations of the dependent variable on the phylogenetic tree. The phylogenetic tree represented the divergence times among taxa based on Irissari et al. (REF) as follows: Divergence times between the taxa within each pair were set to 0.5 million years; divergence between the two *Tropheus* population pairs (*T. moorii* and *T.* sp. black) was set to 1.5 million years ago; divergence between *Tropheus* and *Aulonocara* was set to 5 million years ago, and divergence among samples within taxa was minimal (compare to Fig. 1a).

**Carotenoid extraction and analysis**

Integumentary carotenoids were extracted from some of the same fish as used for RNA sequencing, with skin samples taken from the same body region on other side of the fish.

Skin was sampled from three males per morph and extracted overnight at 4°C in a solution of acetone with butylated hydroxytoluene (BHT, 1 g/L) in volumes ranging from 150 – 1000 µl. The supernatant was recovered and skin samples were extracted overnight at 4°C for a second time. HPLC of skin extracts was carried out in the Agilent 1290 UHPLC System (Agilent Technologies, Waldbronn, Germany) with an Agilent Zorbax Eclipse Plus C18 (2.1 x 50 mm, 1.8 µm Rapid resolution HD, Agilent Technologies Waldbronn, Germany). Mobile phase A consisted of acetonitrile (gradient grade, VWR International, UK), water (18.2 MΩ cm, obtained from a Millipore Milli-Q reference ultrapure water purification system, USA) and formic acid (MS grade, Merck, Germany) in a ratio of 80+20+0.1. Mobile phase B consisted of 2-propanol (HPLC grade, VWR International, UK). A gradient with the following settings was used at a flow rate of 0.50 mL min^-1^: 0-4.5 minutes 0 % B to 100 % B, 4.5 to 5minutes 100 % B, one minute equilibration with 0 % B. An injection volume of 1 µL was used and the column temperature was set to 50°C. The detector recorded an UV spectrum (210-640 nm, 2 nm steps) and the wavelength of 480 nm with a bandwidth of 8 nm with a sampling rate of 40 Hz was recorded. Integrated peak areas (retention time range 1.1 – 4.9 minutes) were related to tissue weight and extraction volume (area x volume / weight) and summed across the two consecutive extracts per sample, to obtain a proxy for comparisons of carotenoid content between skin samples.

**Supplementary Tables and Figures**

**Table S1: Number of RNA sequencing reads obtained for each sample**

| **Sample** | **Raw PE reads** | **Quality trimmed PE reads** |
| --- | --- | --- |
| AhR_1 | 11 698 886 | 11 416 780 |
| AhR_2 | 12 849 011 | 12 655 327 |
| AhR_3 | 11 392 905 | 11 248 242 |
| AhR_4 | 9 837 457 | 9 792 835 |
| AhR_5 | 7 617 960 | 7 582 529 |
| AhR_6 | 9 848 075 | 9 815 306 |
| AbY_1 | 11 433 273 | 11 268 824 |
| AbY_2 | 16 076 683 | 15 876 359 |
| AbY_3 | 9 895 331 | 9 755 585 |
| AbY_4 | 13 245 661 | 13 168 485 |
| AbY_5 | 10 638 293 | 10 581 012 |
| AbY_6 | 7 744 517 | 7 699 719 |
| TbR_1 | 10 189 009 | 10 154 164 |
| TbR_2 | 13 792 672 | 13 611 056 |
| TbR_3 | 16 343 243 | 16 106 230 |
| TbR_4 | 15 026 212 | 14 781 857 |
| TbR_5 | 11 451 969 | 11 364 707 |
| TbR_6 | 11 224 243 | 11 170 763 |
| TbY_1 | 15 030 089 | 14 780 763 |
| TbY_2 | 14 110 544 | 13 934 885 |
| TbY_3 | 11 151 012 | 11 105 105 |
| TbY_4 | 12 488 868 | 12 445 357 |
| TbY_5 | 10 537 157 | 10 497 146 |
| TbY_6 | 12 658 456 | 12 588 406 |
| TmR_1 | 13 648 664 | 13 464 058 |
| TmR_2 | 10 419 572 | 10 277 072 |
| TmR_3 | 11 011 028 | 10 865 503 |
| TmR_4 | 11 374 423 | 11 317 875 |
| TmR_5 | 9 747 323 | 9 714 254 |
| TmR_6 | 12 214 709 | 12 172 822 |
| TmY_1 | 13 267 623 | 13 098 942 |
| TmY_2 | 13 224 763 | 13 034 529 |
| TmY_3 | 13 512 951 | 13 313 438 |
| TmY_4 | 17 043 349 | 16 765 513 |
| TmY_5 | 10 292 921 | 10 257 177 |
| TmY_6 | 9 366 061 | 9 335 854 |

**For Tables S2-S4, see supplementary file 2**

**Table S5: Carotenoid peak area integration. Each skin sample was extracted twice. Areas of UHPLC peaks detected at 480 nm were integrated from 1.1 to 4.9 minutes retention time for each extract (peak area).**

| **color variant** | **extractID** | **peak area** | **extraction volume [µl]** | **skin wet weight [mg]** | **(area/weight)*volume** |
| --- | --- | --- | --- | --- | --- |
| AhR | crude137 | 299.7 | 250 | 10.8 | 6938.08 |
| AhR | crude137_2 | 31.7 | 150 | 10.8 | 440.10 |
| AhR | crude143 | 368.0 | 250 | 13.7 | 6714.62 |
| AhR | crude143_2 | 39.3 | 150 | 13.7 | 429.89 |
| AhR | crude149 | 293.3 | 250 | 14.1 | 5200.83 |
| AhR | crude149_2 | 41.3 | 150 | 14.1 | 438.82 |
| AbY | crude138 | 193.3 | 250 | 10.7 | 4515.26 |
| AbY | crude138_2 | 14.3 | 150 | 10.7 | 200.12 |
| AbY | crude144 | 257.6 | 250 | 16.4 | 3927.27 |
| AbY | crude144_2 | 41.0 | 150 | 16.4 | 375.06 |
| AbY | crude150 | 175.1 | 250 | 10.4 | 4208.49 |
| AbY | crude150_2 | 19.3 | 150 | 10.4 | 277.83 |
| TbR | crude135 | 713.5 | 1000 | 66.6 | 10713.18 |
| TbR | crude135_2 | 93.8 | 500 | 66.6 | 704.29 |
| TbR | crude141 | 406.5 | 1000 | 97.5 | 4168.99 |
| TbR | crude141_2 | 505.5 | 500 | 97.5 | 2592.23 |
| TbR | crude147 | 619.7 | 600 | 32.4 | 11476.22 |
| TbR | crude147_2 | 56.2 | 300 | 32.4 | 520.06 |
| TbY | crude136 | 305.6 | 800 | 57.7 | 4237.13 |
| TbY | crude136_2 | 74.5 | 400 | 57.7 | 516.66 |
| TbY | crude142 | 180.0 | 1000 | 81.6 | 2206.20 |
| TbY | crude142_2 | 116.0 | 500 | 81.6 | 710.96 |
| TbY | crude148 | 276.8 | 800 | 59.6 | 3715.03 |
| TbY | crude148_2 | 52.0 | 400 | 59.6 | 349.19 |
| TmY | crude134 | 723.3 | 600 | 37.4 | 11603.09 |
| TmY | crude134_2 | 68.4 | 300 | 37.4 | 548.85 |
| TmY | crude140 | 628.0 | 800 | 56.4 | 8907.04 |
| TmY | crude140_2 | 67.6 | 400 | 56.4 | 479.45 |
| TmY | crude146 | 827.9 | 600 | 39.9 | 12448.99 |
| TmY | crude146_2 | 67.4 | 300 | 39.9 | 506.90 |
| TmR | crude133 | 666.6 | 300 | 16.5 | 12120.09 |
| TmR | crude133_2 | 63.6 | 200 | 16.5 | 770.55 |
| TmR | crude139 | 1031.2 | 300 | 17.9 | 17281.98 |
| TmR | crude139_2 | 102.0 | 200 | 17.9 | 1140.10 |
| TmR | crude145 | 2710.0 | 300 | 19.1 | 42565.29 |
| TmR | crude145_2 | 204.6 | 200 | 19.1 | 2142.89 |

**Table S6: Differentially expressed genes (RNA-Seq) shared across three pairs of cichlid taxa.**

| **Assembly ID** | **Gene** | **Description** | **logFC** | **logCPM** | **P-Value** | **FDR** |
| --- | --- | --- | --- | --- | --- | --- |
|  |  |  |  |  |  |  |
| **Differential expression AbY vs AhR** | | | | | | |
| TRINITY_DN19772_c0_g3 | urah | Urate (Hydroxyiso-) Hydrolase | -4.78242 | 2.483787 | 9.75E-09 | 1.05E-05 |
| TRINITY_DN20094_c1_g2 | tcaf2 | TRPM8 Channel Associated Factor 2 | 4.727374 | 3.375468 | 4.26E-10 | 7.05E-07 |
| TRINITY_DN22389_c2_g4 | dhrsx | Dehydrogenase/Reductase X-Linked | 1.858147 | 5.322337 | 3.40E-06 | 0.001152 |
| TRINITY_DN30700_c0_g1 | ttc39b | Tetratricopeptide Repeat Domain 39B | 1.410296 | 7.128456 | 0.000364 | 0.037608 |
| TRINITY_DN23236_c2_g1 | nlrc3 | NLR Family CARD Domain Containing 3 | -5.86811 | 2.044929 | 7.00E-06 | 0.00203 |
|  |  |  |  |  |  |  |
| **Differential expression TbY vs TbR** | | | | | | |
| TRINITY_DN19772_c0_g3 | urah | Urate (Hydroxyiso-) Hydrolase | -2.6327 | 5.598374 | 2.94E-13 | 2.25E-10 |
| TRINITY_DN20094_c1_g2 | tcaf2 | TRPM8 Channel Associated Factor 2 | -2.22565 | 2.663853 | 8.18E-09 | 2.83E-06 |
| TRINITY_DN22389_c2_g4 | dhrsx | Dehydrogenase/Reductase X-Linked | -1.28213 | 4.421047 | 0.000139 | 0.01105 |
| TRINITY_DN30700_c0_g1 | ttc39b | Tetratricopeptide Repeat Domain 39B | 6.20009 | 7.07341 | 1.09E-30 | 2.95E-27 |
| TRINITY_DN21087_c0_g4 | nlrc3 | NLR Family CARD Domain Containing 3 | 5.10805 | 0.388476 | 2.82E-06 | 0.00047 |
|  |  |  |  |  |  |  |
| **Differential expression TmY vs TmR** | | | | | | |
| TRINITY_DN19772_c0_g3 | urah | Urate (Hydroxyiso-) Hydrolase | 2.176025 | 5.786274 | 9.29E-07 | 0.000262 |
| TRINITY_DN20094_c1_g2 | tcaf2 | TRPM8 Channel Associated Factor 2 | 2.951413 | 5.330142 | 5.93E-05 | 0.006311 |
| TRINITY_DN22389_c2_g4 | dhrsx | Dehydrogenase/Reductase X-Linked | 2.750947 | 6.022007 | 2.89E-11 | 3.60E-08 |
| TRINITY_DN30700_c0_g1 | ttc39b | Tetratricopeptide Repeat Domain 39B | 2.033236 | 6.718945 | 0.000246 | 0.017848 |
| TRINITY_DN21087_c0_g4 | nlrc3 | NLR Family CARD Domain Containing 3 | 2.038886 | 3.366615 | 2.46E-05 | 0.00329 |

**Table S7: Ranking and statistical analyses of candidate reference genes in the skin samples.**

| **BestKeeper** | | | | **geNorm** | | **NormFinder** | |
| --- | --- | --- | --- | --- | --- | --- | --- |
| **Ranks** | **r** | **Ranks** | **SD** | **Ranks** | **M** | **Ranks** | **SV** |
| *clf2* | 0.982 | *ndufa4* | 0.983 | *cct3* | 0.514 | *clf2* | 0.128 |
| *cct3* | 0.982 | *ywhab* | 0.986 | *clf2* | 0.519 | *cct3* | 0.139 |
| *rab2a* | 0.977 | *rab2a* | 0.990 | *rab2a* | 0.533 | *rab2a* | 0.158 |
| *ndufa4* | 0.971 | *clf2* | 0.995 | *ndufa4* | 0.555 | *ndufa4* | 0.178 |
| *nmrk2* | 0.963 | *ssr2* | 1.019 | *ywhab* | 0.588 | *ywhab* | 0.202 |
| *ywhab* | 0.959 | *cct3* | 1.059 | *ssr2* | 0.601 | *ssr2* | 0.235 |
| *ssr2* | 0.958 | *ppdpf* | 1.065 | *nmrk2* | 0.659 | *nmrk2* | 0.315 |
| *ppdpf* | 0.941 | *cartpt* | 1.120 | *ppdpf* | 0.672 | *ppdpf* | 0.318 |
| *cartpt* | 0.715 | *nmrk2* | 1.207 | *cartpt* | 1.152 | *cartpt* | 0.499 |

Abbreviations: SD = Standard deviation, SV = Stability value, M = Mean stability value.

**Table S8: Phylogenetically controlled ANOVA testing for associations between gene expression levels and skin sample colour.** As dependent variable, we used data from RNA-Seq (TMM normalized read counts) and qPCR (log-transformed RQ values). The independent variable, skin colour, was coded as binary trait (‘yellow’ or ‘red’). Sample size, n = 36.

|  |  | RNA-Seq | | qPCR | |
| --- | --- | --- | --- | --- | --- |
| Gene | df | F | P | F | P |
| *ttc39b* | 1 | 65.02 | **0.005** | 22.96 | **0.026** |
| *dhrsx* | 1 | 11.98 | 0.058 | 20.02 | **0.030** |
| *nlrc3* | 1 | 0.21 | 0.685 | n.a. | n.a. |
| *tcaf2* | 1 | 3.58 | 0.201 | 10.26 | 0.068 |
| *urah* | 1 | 0.05 | 0.851 | 0.043 | 0.576 |

**Table S9: TMM normalized read counts of the five focal genes.**

| Sample | *dhrsx* | *nlrc3* | *tcaf2* | *ttc39b* | *urah* |
| --- | --- | --- | --- | --- | --- |
| TmR_1 | 7,623 | 6,679 | 4,893 | 11,288 | 12,483 |
| TmR_2 | 31,613 | 2,354 | 13,971 | 126,48 | 11,372 |
| TmR_3 | 19,008 | 3,431 | 6,116 | 13,162 | 45,67 |
| TmR_4 | 13,115 | 2,619 | 4,536 | 33,118 | 22,052 |
| TmR_5 | 15,664 | 2,93 | 0,55 | 54,06 | 13,772 |
| TmR_6 | 12,806 | 5,326 | 24,164 | 10,141 | 12,551 |
| TmY_1 | 109,841 | 15,001 | 128,919 | 153,613 | 81,448 |
| TmY_2 | 98,238 | 16,173 | 111,813 | 90,337 | 158,379 |
| TmY_3 | 79,928 | 3,336 | 39,95 | 154,324 | 87,611 |
| TmY_4 | 193,371 | 15,681 | 35,909 | 252,008 | 97,736 |
| TmY_5 | 112,065 | 19,807 | 3,663 | 201,291 | 49,442 |
| TmY_6 | 77,465 | 27,404 | 106,424 | 153,244 | 62,405 |
| TbR_1 | 25,116 | 0 | 5,572 | 1,572 | 82,175 |
| TbR_2 | 42,794 | 0 | 7,514 | 7,506 | 121,684 |
| TbR_3 | 42,138 | 0 | 14,393 | 6,976 | 94,943 |
| TbR_4 | 26,592 | 0 | 11,971 | 3,053 | 66,641 |
| TbR_5 | 21,309 | 0,38 | 11,612 | 1,96 | 70,963 |
| TbR_6 | 21,174 | 0 | 9,748 | 0,475 | 56,642 |
| TbY_1 | 18,753 | 1,56 | 2,038 | 236,693 | 12,017 |
| TbY_2 | 7,64 | 2,922 | 1,055 | 308,853 | 21,244 |
| TbY_3 | 13,488 | 3,883 | 4,035 | 166,49 | 9,06 |
| TbY_4 | 17,53 | 1,941 | 1,649 | 304,763 | 10,847 |
| TbY_5 | 9,112 | 2,296 | 1,424 | 254,3 | 5,207 |
| TbY_6 | 6,476 | 0,268 | 2,616 | 291,481 | 20,243 |
| AhR_1 | 25,326 | 69,798 | 0 | 88,609 | 7,106 |
| AhR_2 | 17,157 | 0 | 0 | 113,988 | 10,623 |
| AhR_3 | 23,002 | 0 | 0 | 76,445 | 8,684 |
| AhR_4 | 14,692 | 60,818 | 0 | 37,696 | 3,802 |
| AhR_5 | 14,884 | 0 | 0,402 | 77,574 | 30,167 |
| AhR_6 | 5,786 | 0 | 0 | 53,17 | 0,853 |
| AbY_1 | 86,046 | 0 | 2,994 | 393,202 | 0,257 |
| AbY_2 | 78,426 | 0 | 1,66 | 164,048 | 0,218 |
| AbY_3 | 73,429 | 0 | 2,255 | 209,553 | 1,316 |
| AbY_4 | 48,877 | 0 | 6,188 | 208,24 | 0,151 |
| AbY_5 | 31,626 | 0 | 0 | 103,2 | 0 |
| AbY_6 | 54,815 | 0 | 13,184 | 138,915 | 0,191 |

**Supplementary Figure S1.** UHPLC chromatograms of carotenoid extracts. Panels show the chromatograms of one typical skin sample per color variant of 1) *T. moorii* “Mbita” (TmY), 2) *T. moorii* “Moliro” (TmR), 3) *T.* sp. ‚black‘ “Ikola” (TbY), 4) *T.* sp. ‚black‘ “Bulu Point” (TbR), 5) *A. baenschi* (AbY) and 6) *A. hansbaenschi* ‚Redflush‘ (AhR). The absorption signal was detected at 480 nm. Peaks after 2.5 minutes correspond to esterified carotenoids (except for free beta carotene at 3.8 min). Exemplary absorption spectra for a red (a) and a yellow (b) carotenoid are given for corresponding HPLC peaks in chromatogram 1.


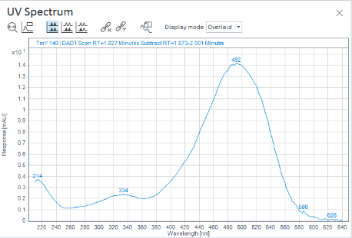


a)


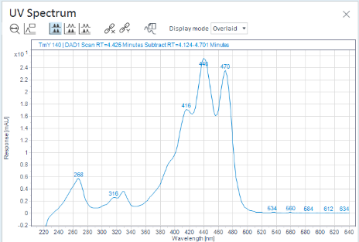


b)

1) TmY

2) TmR

3) TbY

4) TbR

5) AbY

6) AhR

**Supplementary Figure S2.** Top: Integrated peak areas (carotenoid signal) in HPLC analyses of yellow and red skin samples from the cichlid color variants *T. moorii* “Mbita” (TmY), *T. moorii* “Moliro” (TmR), *T.* sp. ‚black‘ “Ikola” (TbY), *T.* sp. ‚black‘ “Bulu Point” (TbR), *A. baenschi* (AbY) and *A. hansbaenschi* ‚Redflush‘ (AhR). The bars represent average values per color variant; open circles represent the individual data points (n = 3 samples per color variant).

Bottom: TMM normalized RNA-Seq reads of *ttc39b* in the yellow and red cichlid color variants (plot design as above, n = 6 samples per color variant).


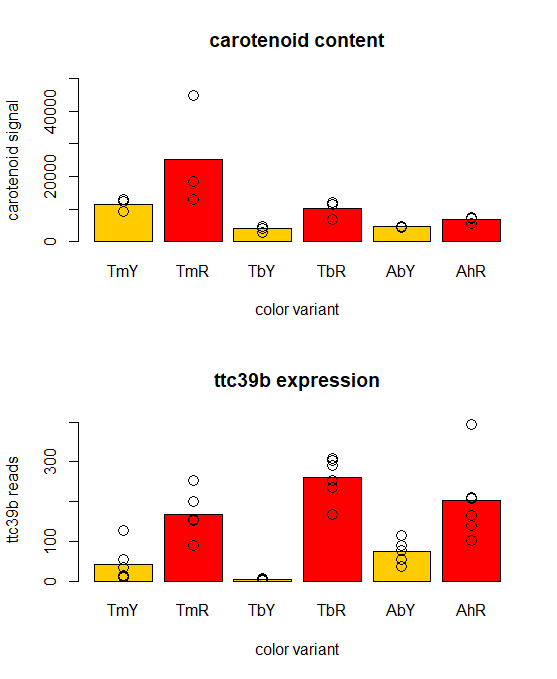


**Supplementary Figure S3.** Volcano plots visualizing differential gene expression within each taxon pair. The cichlid color variants are *T. moorii* “Mbita” (TmY), *T. moorii* “Moliro” (TmR), *T.* sp. ‚black‘ “Ikola” (TbY), *T.* sp. ‚black‘ “Bulu Point” (TbR), *A. baenschi* (AbY) and *A. hansbaenschi* ‚Redflush‘ (AhR). Each dot represents a single transcript, and red dots identify differentially expressed transcripts based on 0.05 FDR.

**
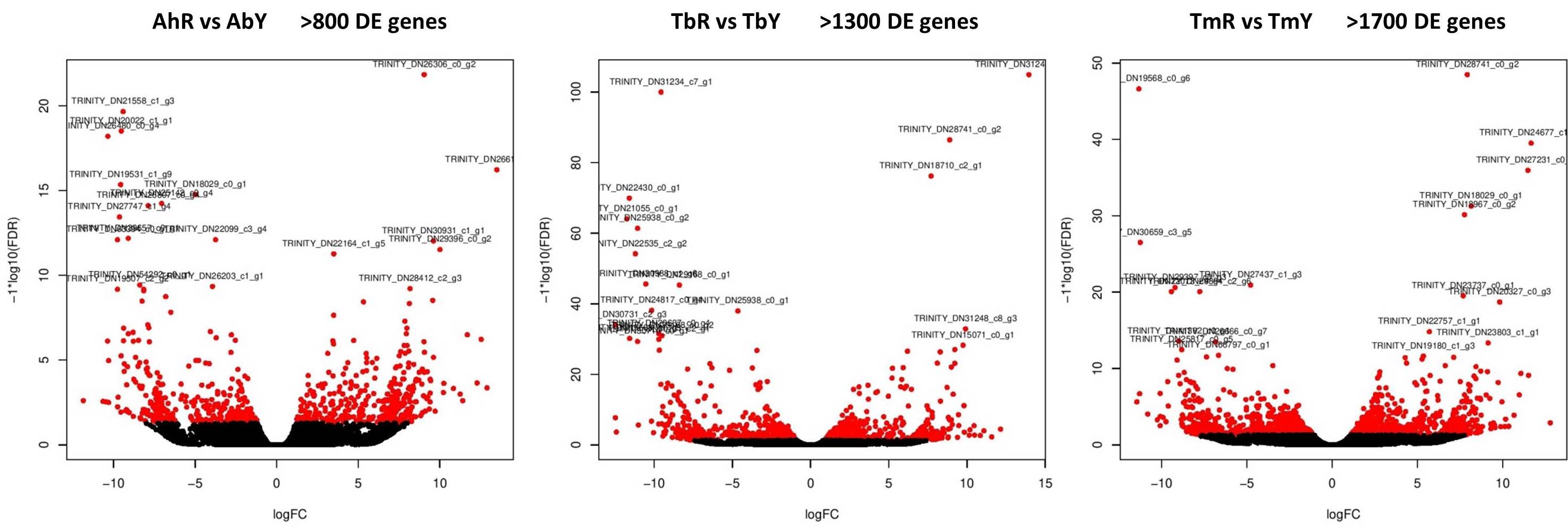
**

References

[1] Bray, N. L., Pimentel, H., Melsted, P. & Pachter, L. 2016 Near-optimal probabilistic RNA-seq quantification. *Nat. Biotechnol.* **34**, 525.

[2] Robinson, M. D. & Oshlack, A. 2010 A scaling normalization method for differential expression analysis of RNA-seq data. *Genome Biol.* **11**, R25.

[3] Robinson, M. D., McCarthy, D. J. & Smyth, G. K. 2010 edgeR: a Bioconductor package for differential expression analysis of digital gene expression data. *Bioinformatics* **26**, 139–140.

[4] Chen, Y., Lun, A. T. L. & Smyth, G. K. 2014 Differential expression analysis of complex RNA-seq experiments using edgeR. In *Statistical analysis of next generation sequencing data*, pp. 51–74. Cham: Springer.

[5] Lun, A. T. L., Chen, Y. & Smyth, G. K. 2016 It’s DE-licious: a recipe for differential expression analyses of RNA-seq experiments using quasi-likelihood methods in edgeR. *Statistical Genomics*, 391–416.

[6] Benjamini, Y. & Hochberg, Y. 1995 Controlling the false discovery rate: a practical and powerful approach to multiple testing. *Journal of the Royal Statistical Society. Series B (Methodological)* **57**, 289–300.

[7] Haas, B. J., Papanicolaou, A., Yassour, M., Grabherr, M., Blood, P. D., Bowden, J., Couger, M. B., Eccles, D., Li, B. & Lieber, M. 2013 De novo transcript sequence reconstruction from RNA-seq using the Trinity platform for reference generation and analysis. *Nature protocols* **8**, 1494.

[8] Altschul, S. F., Gish, W., Miller, W., Myers, E. W. & Lipman, D. J. 1990 Basic local alignment search tool. *Journal of Molecular Biology* **215**, 403–410.

[9] Ahi, E. P., Singh, P., Duenser, A., Gessl, W. & Sturmbauer, C. 2019 Divergence in larval jaw gene expression reflects differential trophic adaptation in haplochromine cichlids prior to foraging. *BMC Evol. Biol.* **19**, 150.

[10] Ahi, E. P., Singh, P., Lecaudey, L. A., Gessl, W. & Sturmbauer, C. 2018 Maternal mRNA input of growth and stress-response-related genes in cichlids in relation to egg size and trophic specialization. *EvoDevo* **9**, 1–17.

[11] Pfaffl, M. W., Tichopad, A., Prgomet, C. & Neuvians, T. P. 2004 Determination of stable housekeeping genes, differentially regulated target genes and sample integrity: BestKeeper–Excel-based tool using pair-wise correlations. *Biotechnol. Lett.* **26**, 509–515.

[12] Andersen, C. L., Jensen, J. L. & Orntoft, T. F. 2004 Normalization of real-time quantitative reverse transcription-PCR data: a model-based variance estimation approach to identify genes suited for normalization, applied to bladder and colon cancer data sets. *Cancer Res.* **64**, 5245–5250.

[13] Vandesompele, J., Preter, K. de, Pattyn, F., Poppe, B., van Roy, N., Paepe, A. de & Speleman, F. 2002 Accurate normalization of real-time quantitative RT-PCR data by geometric averaging of multiple internal control genes. *Genome Biol.* **3**, research0034. 1.

[14] Brawand, D., Wagner, C. E., Li, Y. I., Malinsky, M., Keller, I., Fan, S., Simakov, O., Ng, A. Y., Lim, Z. W. & Bezault, E. *et al.* 2014 The genomic substrate for adaptive radiation in African cichlid fish. *Nature* **513**, 375–381.

[15] Santos, M. E., Baldo, L., Gu, L., Boileau, N., Musilova, Z. & Salzburger, W. 2016 Comparative transcriptomics of anal fin pigmentation patterns in cichlid fishes. *BMC Genomics* **17**, 712.

[16] Singh, P., Börger, C., More, H. & Sturmbauer, C. 2017 The role of alternative splicing and differential gene expression in cichlid adaptive radiation. *Genome Biology and Evolution* **9**, 2764–2781.

[17] Hellemans, J., Mortier, G., Paepe, A. de, Speleman, F. & Vandesompele, J. 2007 qBase relative quantification framework and software for management and automated analysis of real-time quantitative PCR data. *Genome Biol.* **8**, R19.

[18] Ramakers, C., Ruijter, J. M., Deprez, Ronald H. Lekanne & Moorman, A. F. M. 2003 Assumption-free analysis of quantitative real-time polymerase chain reaction (PCR) data. *Neurosci. Lett.* **339**, 62–66.

[19] Pfaffl, M. W. 2001 A new mathematical model for relative quantification in real-time RT–PCR. *Nucleic Acids Res.* **29**, e45-e45.
